# Incorporating peak grouping information for alignment of multiple liquid chromatography-mass spectrometry datasets

**DOI:** 10.1093/bioinformatics/btv072

**Published:** 2015-02-02

**Authors:** Joe Wandy, Rónán Daly, Rainer Breitling, Simon Rogers

**Affiliations:** ^1^School of Computing Science, University of Glasgow, Glasgow, UK, ^2^School of Computing and Mathematical Sciences, Liverpool John Moores University, Merseyside, UK and ^3^Manchester Centre for Synthetic Biology of Fine and Speciality Chemicals (SYNBIOCHEM), Manchester Institute of Biotechnology, University of Manchester, Manchester, UK

## Abstract

**Motivation:** The combination of liquid chromatography and mass spectrometry (LC/MS) has been widely used for large-scale comparative studies in systems biology, including proteomics, glycomics and metabolomics. In almost all experimental design, it is necessary to compare chromatograms across biological or technical replicates and across sample groups. Central to this is the peak alignment step, which is one of the most important but challenging preprocessing steps. Existing alignment tools do not take into account the structural dependencies between related peaks that coelute and are derived from the same metabolite or peptide. We propose a direct matching peak alignment method for LC/MS data that incorporates related peaks information (within each LC/MS run) and investigate its effect on alignment performance (across runs). The groupings of related peaks necessary for our method can be obtained from any peak clustering method and are built into a pair-wise peak similarity score function. The similarity score matrix produced is used by an approximation algorithm for the weighted matching problem to produce the actual alignment result.

**Results:** We demonstrate that related peak information can improve alignment performance. The performance is evaluated on a set of benchmark datasets, where our method performs competitively compared to other popular alignment tools.

**Availability:** The proposed alignment method has been implemented as a stand-alone application in Python, available for download at http://github.com/joewandy/peak-grouping-alignment.

**Contact:** Simon.Rogers@glasgow.ac.uk

**Supplementary information:** Supplementary data are available at *Bioinformatics* online.

## 1 Introduction

Liquid chromatography, coupled to mass spectrometry (LC/MS) is one of the most widely used techniques in untargeted proteomic and metabolomic studies (Vandenbogaert *et al.*, 2008). In proteomic or metabolomic experiments, the input sample to the LC/MS instrument is a complex mixture of peptides or metabolites. Compounds in the mixture are separated in time through liquid chromatography (LC) and subjected to mass spectrometry (MS) analysis. The result of this process is a mass chromatogram: an intensity surface across the mass-to-charge ratio (*m*/*z*) and retention time (RT) dimensions. From this surface, it is possible to extract individual peaks (corresponding to ions in the mass spectrometry). In this article, we use the term ‘run’ to refer to the output from running any biological sample through the LC/MS instrument once, and ‘feature’ to refer to a tuple of (m/z,RT) values derived from a single peak.

Experiments in biology involve a comparison of multiple samples, so a typical LC/MS dataset consists of data from several runs. In order to compare peaks across these runs, they have to be matched, and while the measured *m*/*z* of a particular peak tends to be conserved in high-precision mass spectrometry, the RT is prone to drifting. These RT shifts can be highly nonlinear (Podwojski *et al.*, 2009) and are the result of instrument-specific factors, such as the condition of the chromatographic column, gradient slope, and temperature (Christin *et al.*, 2008), or experiment-specific factors, such as instrument malfunctions or columns that need be replaced mid-experiment. Due to this RT variation, a single peak from one run can have several potential matches in another run. The problem of matching peaks and correcting RT shifts is broadly referred to as *alignment*. Errors during the alignment can have a detrimental effect on the subsequent analysis.

In a comprehensive review, Smith *et al.* (2013) identify two broad alignment approaches: warping and direct matching. In the warping approach, an alignment tool seeks to fit an RT correction function (typically a regression model) between runs. Once the RT shifts have been corrected, the correspondence of peaks can be found through any method that matches peak features across runs. Early warping approaches, such as dynamic time warping (Sakoe and Chiba, 1978), correlation optimized warping (Nielsen *et al.*, 1998) and parametric time warping (Eilers, 2004), are predominantly based on dynamic programming, and use only the time information present in the Total Ion Chromatograms (TIC), but recent warping approaches have included the *m*/*z* dimension as well (Christin *et al.*, 2008). In the alternative approach of direct matching, the goal of alignment is to skip the warping step and directly match peaks across runs. Direct approaches therefore require that the peak (i.e. feature) extraction step has already been completed. The majority of direct matching approaches consist of two stages: computing feature similarity and using this similarity to match the features. A wide range of feature similarity measures have been proposed to compare the *m*/*z* and RT values of two peaks, including normalized weighted absolute difference (Pluskal *et al.*, 2010), cosine similarity (Hoffmann *et al.*, 2012), Euclidean distance (Ballardini *et al.*, 2011) and Mahalanobis distance (Voss *et al.*, 2011). Once similarity has been computed, feature matching can be established through either a greedy or combinatorial matching method. Since matching across all runs at once can be computationally expensive (due to the exponential growth of features to be considered), complete multiple alignment results are usually produced through some merging scheme of pair-wise runs.

Greedy feature matching methods work by making a locally optimal choice at each step, in the hope that this will lead to an acceptable matching solution in the end. RTAlign in MSFACTs (Duran *et al.*, 2003) merges all runs and greedily groups features into aligned peaksets within a user-defined RT tolerance. Join Aligner (Pluskal *et al.*, 2010) in MZmine2 merges successive runs to a master peaklist by matching features greedily according to their similarity scores within user-defined *m*/*z* and RT windows. Similarly, MassUntangler (Ballardini *et al.*, 2011) performs nearest-distance matching of features, followed by various intermediate filtering and conflict-resolutions steps. Recent advances in direct matching methods have also posed the matching task as a combinatorial optimization problem. Simultaneous Multiple Alignment (SIMA) (Voss *et al.*, 2011) uses the Gale-Shapley algorithm to find a stable matching in the bipartite graph produced by joining peaks (nodes) from one run with peaks from another run that are within certain *m*/*z* and RT tolerances. Wang and Lam (2013) explore the application of the classical Hungarian algorithm to find the maximum weighted bipartite matching. Bidirectional best hits peak assignment and cluster extension (BIPACE) (Hoffmann *et al.*, 2012) establishes correspondence by finding the maximal cliques in the graph. SMFM (Lin *et al.*, 2013) uses dynamic programming to compute a maximum bipartite matching under a relaxed bijective mapping assumption for time mapping.

**Fig. 1.**
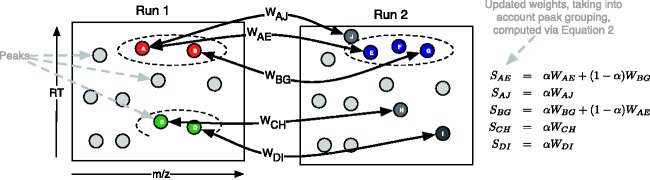
Illustrative example of the incorporation of grouping information into the similarity score. Each node in the figure is a peak feature, and dotted ovals represent groups of related peaks, e.g. isotopes, fragments, etc. Initially weights (e.g. *W_AE_*) are computed for pairs of peaks (one from each run) with *m*/*z* and RT within pre-defined thresholds. These weights are converted into an overall score by incorporating grouping information. For example, peak pairs (*A*, *E*) and (*B*, *G*) are both within the threshold. As *A* and *B* are in the same group, and *E* and *G* are in the same group, the weights between pairs (*A*, *E*) and (*B*, *G*) are upweighted. Peak *J* is not related to any peaks that could be matched with *A*’s related peaks and the similarity between *A* and *J* is therefore downweighted (because α≤1). The same applies to similarities between pairs (*C*, *H*) and (*D*, *I*)

Many of the tools surveyed in Smith *et al.* (2013) make the assumption that elution order of peaks is preserved across runs. Often, a tool also has a number of user-defined parameters, varying which can drastically change the alignment. More importantly, none of the tools surveyed in Smith *et al.* (2013) take into account the structural dependencies between coeluting peaks when solving the correspondence problem. Such information could potentially be used to improve the alignment process, since a set of coeluting peaks (derived from the same compound/peptide fragment) in one run should generally be aligned to another set of coeluting peaks in the other run. In this work, we propose the inclusion of *related* *peak* information into the matching process. We define related peaks to be all those peaks that appear in a run due to the presence of one compound in the sample being analyzed. Examples of related peaks are isotope peaks, multiple adduct and deduct peaks, and fragment peaks (Scheltema *et al.*, 2009). Such peaks should coelute from the column and have similar chromatographic shapes. Our proposed approach uses information as to which peaks are related to which other peaks in an individual run, to modify peak to peak similarity scores across runs. The related peak information can come from any peak grouping (e.g. clustering via RT) method. Our key assumption is that groups of co-eluting peaks will be preserved across runs. The idea is illustrated in Figure 1. In the figure, initial weights are computed between pairs of peaks in the two runs that are within *m*/*z* and RT tolerances (e.g. *W_AE_* and *W_AJ_*). When related peak information is added, the similarity between peaks *A* and *E* is increased due to peak *A* being related to another peak (*B*) that is similar to a peak (*G*) related to *E*. On the other hand, the similarity between *A* and *J* is not increased as *J* does not have any related peaks that could potentially be matched to peaks related to *A*. In other words, we are proposing using the *structural dependencies* present between peaks in each run to modify the similarity scores and improve alignment performance: the more peaks related to *A* that could be matched to peaks related to *E*, the more likely it becomes that *A* should be matched to *E*.

## 2 Materials and methods

### 2.1 Direct matching method

Our proposed alignment method combines a novel similarity score with maximum weighted bipartite matching. This results in pair-wise alignments which can be, if desired, extended to multiple alignments with hierarchical merging strategy. In such merging strategies, having an accurate initial pair-wise alignments is important because of its influence on the final multiple alignment results. In the following sections, we describe each step in more detail.

### 2.2 Feature similarity

Suppose we wish to align run A containing *N_A_* peaks with run B containing *N_B_* peaks. We follow SIMA (Voss *et al.*, 2011) in using the Mahalanobis distance between two peaks $p_{i}\in A,\;p_{j}\in B$ where each peak is a vector of its *m*/*z* and RT values $p_{i}={\left[m_{i},t_{i}\right]}^{T}$ and $p_{j}={\left[m_{j},t_{j}\right]}^{T}$. The distance is given as:

$$D(p_{i},p_{j})=\sqrt{{\left(p_{i}-p_{j}\right)}^{T}\Sigma^{-1}(p_{i}-p_{j})},$$

where the covariance matrix Σ is a diagonal matrix of mass-to-charge tolerance $\sigma_{m}^{2}$ and retention time tolerance $\sigma_{t}^{2}$. The diagonal covariance matrix Σ assumes independence between the $\sigma_{m}^{2}$ and $\sigma_{t}^{2}$ components. To reduce the computational burden, entries in ***D*** are only computed when the peaks’ *m*/*z* and RT values are within *σ_m_* and *σ_t_*. We now define the similarity score between two peaks as one minus their normalized distance:

(1) $$W(p_{i},p_{j})=1-\frac{D(p_{i},p_{j})}{D_{max}},$$

where *D_max_* is the maximum computed distance between peaks in the two runs being aligned. Collectively, we call the $N_{A}\times N_{B}$ matrix of similarity scores between all peaks in run A and B to be ***W***.

### 2.3 Incorporating related peak groups

The similarity score matrix **W** can now be combined with related peak information to obtain a final score, **S**:

(2) S=αW+(1−α)L

where ***L*** is the cluster similarity score between the two peaks in a single run (described below), and *α* (0≤α≤1) is a parameter controlling the relative influence of the two components. To compute ***L***, we require related peak groupings from the two runs being aligned. This takes the form of an $N_{A}\times N_{A}$ matrix $C^{A}$ for run A and an $N_{B}\times N_{B}$ matrix $C^{B}$ for run B. Entries in $C^{A}$ and $C^{B}$ can be either binary (0, 1) or probability values, depending on the peak grouping algorithm used. For example, if a greedy clustering approach is applied to the features in run A, the *ij*-th element of $C^{A}$ will be either 1 or 0, depending on whether the *i*-th and *j*-th features (peaks) in A are clustered together (1) or not (0). Note that in the following, we define the diagonal components of both matrices to be zero to avoid double counting. We then compute **L** as follows:

(3) $$L=C^{A}\cdot W\cdot C^{B}.$$

The resulting matrix gives cluster similarity scores such that each element *L_ij_* of ***L*** is the sum of weight from peaks in the same cluster as *i* in run *A* to peaks in the same cluster as *j* in run *B*. This allows us to use the matrix ***L*** to upweight the similarity scores between peaks in the same cluster in one run that also have more potential matches to peaks in the same cluster in the other run of the matching. Computation of Equation 3 is illustrated in Figure 1. The ratio parameter *α* controls how much clustering information we bring into the overall similarity score matrix ***S***, with its value bounded in 0≤α≤1. Setting *α* = 1 results in a matching that uses only information from ***W***, the similarity score matrix. Setting *α* = 0 means that the matching is performed based only on the cluster similarity score ***L***. From our experience, a reasonable range of values for *α* lies between 0.2 and 0.4.

Our proposed approach is independent of the method used to group related peaks in each run. For comparison, we call our method that does not use the cluster similarity score (*α* = 1) to be Maximum-Weighted (MW). We demonstrate the performance improvement from incorporating related peaks information using two different clustering algorithms: a greedy RT clustering approach Maximum-Weighted-Greedy (MWG) and a statistical mixture model [Maximum-Weighted-Mixture (MWM)]. MWG starts with the most intense peak in the dataset and clusters it with other candidate peaks inside a retention time window *g*_tol_. The next most intense peak that has not already been clustered is processed, and the grouping process is repeated until all peaks are exhausted. If chromatographic peak shapes information is available (such as for the Metabolomic dataset used in Section 4.2), the Pearson correlation coefficient between the chromatographic peak signals of the most intense peak and the candidate peaks are computed. Only candidate peaks with Pearson correlation values greater than some threshold *c* are accepted into the newly-formed cluster. This greedy clustering process results in binary grouping matrices $C^{A}$ and $C^{B}$. MWM uses an infinite Gaussian mixture model on RT (see e.g. Rasmussen, 2000). Analytical inference is not possible in this model, so a Gibbs sampling procedure is used to sample clusterings used to compute the probability of two features (peaks) to be in the same cluster. These probabilities comprise the elements of $C^{A}$ and $C^{B}$, i.e. the *ij*th element of $C^{A}$ is the proportion of samples from run A in which peaks *i* and *j* were in the same cluster. More details of the mixture model and sampling procedure are provided in the Supplementary Material.

### 2.4 Feature matching

Alignment between two runs can be represented as a matching problem on a bipartite graph *G*, where nodes in the graph are the features, edges are the potential correspondence between features and the weights on the edges are the similarity scores (entries in *S*) between features. In SIMA (Voss *et al.*, 2011), the Gale–Shapley algorithm (Gusfield and Irving, 1989) is used to find a stable matching in *G*. A matching is stable if there are no two features in different runs that would prefer to be matched to each other than to their currently matched partners. Since the stable matching is computed based on ranked preference, valuable information could be discarded as distances between features are converted to ranks. As such, we prefer to use a method that maximizes the total sum of similarity scores of matched features (maximum weighted matching).

The benefit of maximum weighted bipartite matching in solving the peak correspondence problem has been studied in Wang and Lam (2013) in their LWBMatch tool. LWBMatch shows that such matching method, coupled to a local regression method, is able to align runs having large and systematic drifts in RT values. The well-known Hungarian algorithm (Kuhn, 1955) attributed to Kuhn and Munkres is used in LWBMatch to solve this problem. The time complexity of the Hungarian algorithm is $O(n^{3})$, where *n* is the number of peaks in the larger set. While the Hungarian algorithm’s implementation can be improved to $O(n^{2}log\;n)$ by using Fibonacci heaps for the shortest path computation, the polynomial time complexity required in this scheme is often too slow to be practical for alignments of the large number of runs produced in large-scale untargeted LC-MS studies. Consequently, we compute an approximation of the maximum weighted matching using a simple greedy algorithm that runs in O(m log n) time, where *n* and *m* denote the number of vertices and edges in the bipartite graph *G* to be solved. The greedy algorithm is straightforward to describe: pick the heaviest edge *e* in *G*, where *e* represents a potential match between nodes (features). Add *e* to the matching solution *M* and remove all other edges adjacent to *e* from *G*. Repeat until all edges in *G* have been exhausted. This simple greedy algorithm is known to provide a lower bound of at least 1/2 of the maximum weight in the matching (Duan *et al.*, 2011).

### 2.5 Evaluation datasets

Performance of the proposed methods and other benchmark methods is evaluated on LC-MS datasets from proteomic, metabolomic and glycomic experiments. The proteomic datasets are obtained from Lange *et al.* (2008) while the glycomic dataset comes from Tsai *et al.* (2013). These datasets provide the ground truth for alignment and have used to benchmark alignment performance in other evaluation studies (Ballardini *et al.*, 2011; Lange *et al.*, 2008; Pluskal *et al.*, 2010; Tsai *et al.*, 2013; Voss *et al.*, 2011). Additionally, we also introduce a metabolomic dataset generated from the standard runs used for the calibration of chromatographic columns (Creek *et al.*, 2011). The runs were produced from different LC-MS analyses separated by weeks, representing a challenging alignment scenario. Further details on each dataset and the construction of alignment ground truth can be found in Section 2 of the Supplementary Material.

## 3 Evaluation study

### 3.1 Experimental setup

The alignment tools evaluated have in common user-defined *m*/*z* and RT window parameters. These parameters act as hard thresholds that determine the solution space to be explored in the *m*/*z* and RT dimensions when matching features. Performance of all alignment procedures is highly dependent on the assumptions and choice of parameter values that underpin them (Smith *et al.*, 2013). For example, warping methods must make assumptions regarding the mathematical form of the warping function and are dependent on a good choice of reference run. Direct matching approaches typically need to decide on the form of peak similarity function, and define some *m*/*z* and RT windows, outside of which, peaks cannot be matched. While the *m*/*z* window and parameters can often be determined based on the mass accuracy of the measurement equipment, there is no obvious way to determine the RT window and associated parameters. The optimal choice of such parameters could have a significant influence on the final results (Smith *et al.*, 2013), and there is no reason to believe that these parameters should remain constant across different experiments.

Previous studies on the proteomic and metabolomic datasets presented here (Ballardini *et al.*, 2011; Lange *et al.*, 2008; Voss *et al.*, 2011) varied the window parameters and reported the best performance achieved. While informative, this procedure is unrealistic due to the role of the ground truth in choosing the optimal parameter values. To provide a more realistic estimate of performance, we also present the performance on a separate testing set. In other words, we optimize the window parameters on one alignment task and report the performance when using these optimized parameters on a second task (distinct from the first task). This reflects the scenario where the parameters are set based on performance on a previous dataset or due to information supplied from the instrument manufacturer and tells us how critical setting these parameters is for each method.

In this article, *training set* refers to the data on which alignment parameters are optimized and *testing set* refers to the independent set on which alignment performance is evaluated. We believe that this represents a more realistic measure of alignment performance and provides us with some information as to how the different algorithms generalize to new datasets. We addressed the lack of comparative evaluation of alignment tools as discussed in Smith *et al.* (2013) by independently reproducing key results from Lange *et al.* (2008) and Voss *et al.* (2011) for the Join and SIMA alignment methods. Our evaluation studies were performed on datasets selected in Section 2.5 to validate the hypothesis that using related peak information can improve alignment performance. Since most direct matching algorithms work in a pair-wise fashion (pairs of runs are matched and the results combined), pair-wise performance therefore limits overall performance, justifying the choice for our experiments. For the proteomic datasets, each fraction in P1 has two runs used for alignment, while each fraction in P2 has three runs (we use only the first two to establish pair-wise alignments). Similarly for the metabolomic and glycomic datasets, we randomly extracted 30 pairs of runs for training and another 30 pairs of runs for testing performance evaluation.

Performance is evaluated in terms of precision, recall and F_1_-score. Looking at pair-wise matching, we can define the following positive and negative instances with respect to some pair-wise alignment ground truth:

- True Positive (***TP***): pairs of peaks that should be aligned and are aligned.
- False Positive (***FP***): pairs of peaks that should not be aligned but are aligned.
- True Negative (***TN***): pairs of peaks that should not be aligned and are not aligned.
- False Negative (***FN***): pairs of peaks that should be aligned but are not aligned.

In the context of alignment performance, precision ($\frac{\mathrm{TP}}{\mathrm{TP}+\mathrm{FP}}$) is the fraction of aligned pairs in the output that are correct with respect to the ground truth, while recall ($\frac{\mathrm{TP}}{\mathrm{TP}+\mathrm{FN}}$) is the fraction of aligned pairs in the ground truth that are aligned in the output. A perfect alignment would have both precision and recall to be 1. In addition, we also computed the F_1_ score (the harmonic mean of precision and recall) such that $F_{1}=2(precision\cdot recall)/(precision+recall)$. Only feature pairs present in the ground truth are considered for evaluation. The idea of using pair-wise matching to define alignment performance evaluation is not new, and has also been done in Wang and Lam (2013). Collectively for the purpose of performance evaluation, the set of Precision, Recall and F_1_ values is referred to as a ‘measurement’.

### 3.2 Other alignment tools for comparison

Our proposed approach was benchmarked against MZmine2’s Join Aligner (Pluskal *et al.*, 2010) and SIMA (Voss *et al.*, 2011). These tools employ different approaches towards alignment. Join Aligner is a greedy direct-matching method, while SIMA is a combinatorial direct-matching method, with an optional warping step to correct RT shifts after an initial matching has been established.

#### 3.2.1 MZmine2’s join aligner

Users of the MZmine2’s toolkit may have good reasons to prefer Join Aligner to the more recent RANSAC Aligner due to its simplicity and speed. Join Aligner produces a deterministic alignment output (so running it each time on the same input and parameters gives the same result), in contrast to the RANSAC aligner, which is non-deterministic. Join Aligner has relatively few parameters to configure, the most important ones being the *m*/*z tolerance* and *retention time tolerance* parameters. These parameters are used for thresholding and score calculations, and they were varied within reasonable ranges during our experiments.

#### 3.2.2 Simultaneous multiple alignment

The two most important parameters used in SIMA for thresholding and computing feature similarities are the $T_{\left(m/z\right)}$ and *T_rt_* parameters (equivalent to our *σ_m_* and *σ_t_*). We let these two parameters vary in our experiments. SIMA also offers an optional step to correct for retention time distortion by constructing a smooth and monotonic warping function for the maximum likelihood alignment path after the initial matching has been done. The utility of this optional step is not obvious to end-users, since it requires additional parameters to configure and relies on having an initial correspondence established. Therefore, we chose to test only the core matching functionality in SIMA.

## 4 Results

We conducted several experiments on the proteomic, metabolomic and glycomic datasets, each designed to test a different aspect of alignment tools’ performance. Details on the parameter optimizations for evaluated tools are provided in the Supplementary document.

### 4.1 Proteomics experiments

#### 4.1.1 Single-fraction experiment

Both P1 and P2 data consist of multiple fractions. In the first experiment, we investigate the best possible performance by using the same fraction as training and testing sets. On each training set (a fraction), we optimized the *m*/*z* and RT window parameters for alignments. The *m*/*z* parameters are in parts per million, normally notated ‘ppm’ and the range of *m*/*z* parameters used were {1.0,1.1,...,2.0} and RT {5,10,...,300} seconds. Parameters that control the grouping and influence of the cluster similarity score for our MWG and MWM methods were also optimized. The ratio parameter *α* was set to {0.1,0.2,...,1} for both MWG and MWM. The grouping tolerance *g_tol_* was set to {1,2,...,10} seconds for greedy clustering, while the same hyperparameters were used for clustering of all fractions in case of mixture-model clustering (further details on parameter range selections are in the Supplementary Material).

**Fig. 2.**
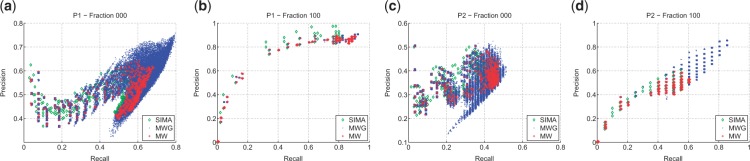
Precision and recall training performance for all parameters (*m*/*z*, RT tolerance, *α* and *g_tol_*) varied in the experiment for the fractions containing the most (Fig. 2**a** and **c**) and least (Fig. 2**b** and **d**) number of features in the P1 and P2 datasets. Plots for all the remaining fractions can be found in Figures 1 and 2 of Supplementary Material

The results are shown in Tables 1 and 2 (full results, including precision and recall values, can be found in the Supplementary Material). We see that approximate maximum weighted matching (MW) alone performs competitively to other tools. On the P1 data (Table 1), incorporating grouping information (MWG, MWM) improves F_1_ score performance over MW. MWG outperforms MWM, which may be due to the fact that the greedy approach is easier to optimize. For the P2 data (Table 2), which contains features with significantly higher RT drift across runs, again we find that MW is competitive and clustering information (MWG) improves performance for all fractions. The results here show the potential of our proposed approach: any peak grouping results expressed in a suitable matrix format can be incorporated into our method, and used as additional information during the matching stage. Figure 2 shows how the benefit of incorporating clustering information is realized during matching: it allows the matching methods to explore regimes in the solution space having higher precision and recall values. On some training fractions, both methods that incorporate clustering information show significant increases in the best possible F_1_ score. For dataset P1 fraction 000, this is an 11% improvement for MWG and a 7.5% improvement for MWM. For dataset P2 fraction 100, this is a 51% improvement for MWG and 25% improvement for MWM. Smaller improvements can be observed from other fractions in the Proteomic datasets too. The full results for all fractions, including computed precision and recall values, are available in the Supplementary document.

**Table 1.** F_1_ scores for the single-fraction experiment results on the P1 dataset

Fraction	Join	SIMA	MW	MWG	MWM
000	0.63	0.64	0.64	**0.77**	0.71
020	0.88	0.88	0.88	**0.95**	0.90
040	0.82	0.83	0.85	**0.87**	0.86
060	0.76	0.78	0.78	**0.88**	0.83
080	0.90	0.89	0.88	**0.92**	0.90
100	0.89	0.89	0.89	**0.91**	0.91
Mean	0.81	0.82	0.82	**0.88**	0.85

*Notes:* The tool with the highest F_1_ score for each fraction is highlighted in bold. The results for ‘All’ show the average F_1_ scores of individual fractions.

**Table 2.** F_1_ scores for the single-fraction experiment results on the P2 dataset

Fraction	Join	SIMA	MW	MWG	MWM
000	0.45	0.45	0.45	**0.49**	0.45
020	0.77	0.78	0.79	**0.80**	0.79
040	0.77	0.78	0.77	**0.80**	0.77
080	0.66	0.68	0.67	0.67	**0.72**
100	0.55	0.58	0.56	**0.85**	0.70
Mean	0.64	0.65	0.65	**0.72**	0.69

*Note:* The tool with the highest F_1_ score for each fraction is highlighted in bold. The results for ‘All’ show the average F_1_ scores of individual fractions.

#### 4.1.2 Multiple-fractions experiment

The single-fraction experiment does not represent a very realistic scenario as the optimal parameters were determined with respect to an alignment ground truth; practitioners might not possess that information in real analytical situations. In this experiment, we improved upon the single-fraction experiments by using each fraction in each dataset as the training set and the remaining fractions as the testing set. Parameters were optimized on the training set and performance evaluations were performed on the testing set. This training–testing procedure produces six measurements for P1 and five measurements for P2, corresponding to the number of training fractions in each dataset. The overall F_1_ score reported for each measurement is the average F_1_ scores from individual testing fractions. The aim of this experiment is to investigate how well the different methods generalize to data that may have slightly different characteristics from that used to optimize the parameters—i.e. how critical the particular parameter values are.

Tables 3 and 4 show the F_1_ score across measurements (full results in the Supplementary Material). On P1, the best overall performance is achieved by our methods that incorporate clustering information into alignment (MWG, MWM). On P2, the results are less homogeneous, with no method consistently performing best on all the different testing fractions. The implication is discussed in Section 5.

**Table 3.** Multiple-fractions experiment results for the P1 dataset

Training Frac.	Testing performance				
	Join	SIMA	MW	MWG	MWM
000	0.82	0.85	0.82	**0.86**	**0.86**
020	0.78	0.76	0.78	**0.79**	0.75
040	0.78	0.76	0.77	0.79	**0.81**
060	0.78	0.78	0.77	**0.84**	0.83
080	0.71	0.73	0.72	0.77	**0.78**
100	0.75	0.77	0.74	0.76	**0.78**

*Note:* For each training fraction, the reported testing performance is the average of individual F_1_ scores from the testing fractions. The top-performing method (highest F_1_ score) is highlighted in bold.

**Table 4.** Multiple-fractions experiment results for the P2 dataset

Training fraction	Testing performance				
	Join	SIMA	MW	MWG	MWM
000	0.62	**0.64**	0.61	0.48	0.61
020	**0.58**	0.56	0.55	0.43	0.55
040	0.52	**0.56**	**0.56**	0.41	**0.56**
080	0.56	0.50	0.50	0.50	**0.57**
100	**0.63**	0.57	0.56	0.44	0.57

*Notes:* For each training fraction, the reported testing performance is the average of individual F_1_ scores from the testing fractions. The top-performing method (highest F_1_ score) is highlighted in bold.

### 4.2 Metabolomic and glycomic datasets

We further explore the performance of our proposed methods on the metabolomic and glycomic datasets. From the full dataset, we randomly extracted 30 pairs of runs as the training sets and another 30 pairs of runs as the testing sets. Each training set is paired to a testing set. Parameters were optimized on the training set and the best attainable performance reported as the training performance. Generalization performance is evaluated on testing sets using the optimal parameters from the training stage.

**Fig. 3.**
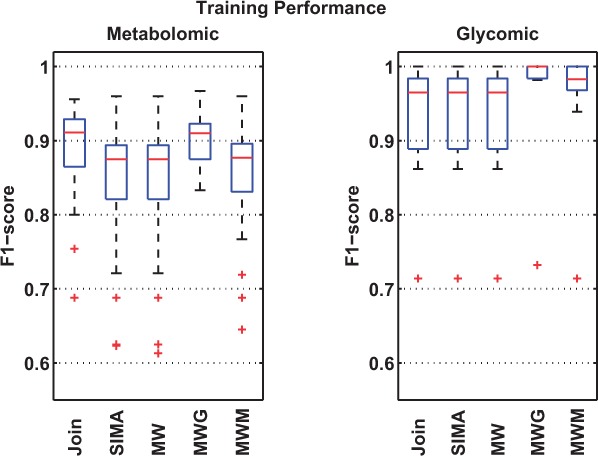
Training performance shows the best F_1_ scores obtained by each method on 30 pairs of randomly selected metabolomic and glycomic training sets

**Fig. 4.**
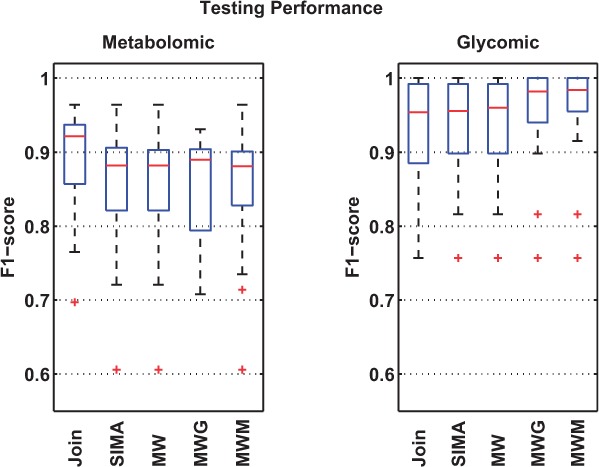
Testing performance shows how well each method generalize on the 30 different testing sets, each evaluated using the optimal training parameters from its corresponding training set

Figures 3 and 4 summarize the results from the experiments (detailed full results and parameter range selections are described in the Supplementary Material). We see that all methods perform better on the glycomic set than on the metabolomic set. This is explained by the fact that the metabolomic runs represent a generally more challenging alignment scenario with significantly more features to align. MW performs identically to SIMA on both datasets due to the similar form of Mahalanobis distance function used. This is despite the differences in the actual matching method that establishes feature correspondences in SIMA and MW. On the glycomic dataset, adding clustering information improves the training performance, with an increase in the mean of the F_1_ scores across 30 measurements from 0.89 (MW) to 0.93 (MWG) and 0.92 (MWM). This also translates into statistically significant improvements on the testing sets for both MWG (*p* = 0.01, paired *t*-test) and MWM (*p* = 0.002, paired *t*-test) over MW.

On the metabolomic dataset, where it is potentially harder to produce good clustering results due to the larger number of peaks and the more complex elution profile, we observe improvements in the mean of the F_1_ scores from 0.83 (MW) to 0.90 (MWG) and 0.85 (MWM) on the training sets. These are also statistically significant improvements for both MWG (*p* < 0.001, paired *t*-test) and MWM (*p* < 0.001, paired *t*-test) over MW. The training results confirm our hypothesis that indeed incorporating clustering information (by modifying the similarity matrix used for matching in the proposed manner) can be used to help improve matching results over the case when such information is not used. However, this does not translate into any statistically significant improvements on the testing sets, suggesting that for the metabolomic dataset evaluated here, our proposed methods are also sensitive to parameter choices, and the choices of particular parameters (especially for the clustering step) that work on some runs may not generalize well to others. Note that unlike in the Proteomic and Glycomic experiments, the results for MWG shown here (also referred to as MWG(RT + PS) in Section 3.4 of the Supplementary Material) takes into account the Pearson correlations of the chromatographic shapes between peak features during the clustering process. Results for MWG that consider only the RT values (referred to as MWG(RT) in the Supplementary) for grouping of related peaks can be found in Section 3.4 of the Supplementary Material.

## 5 Discussion and conclusion

In this article, we have proposed a novel peak matching method that incorporates related peak information to improve alignment performance. The method takes related peak information in the form of peak-by-peak binary or real-valued similarity matrices and as such is independent of the particular method used to compute these. The method fits into the category of *direct matching* approaches—those alignment approaches that do not perform an explicit time-warping phase. Our experimental results demonstrate the potential of this approach. From the training results, we see evidence of performance improvement across all evaluated datasets by incorporating grouping information into the matching process in the proposed manner. With the exception of the metabolomic dataset, both the greedy and model-based clustering approaches evaluated in our experiments rely only on the RT information for grouping related peaks. In the case of the noisiest data (dataset P2 fraction 000), we observe some combinations of parameters that result in training points with reduced precision and recall values. These are likely due to the difficulty of producing a high-quality grouping of related peaks with sub-optimal parameters especially when only the RT information is used. Comparisons of matching performances on the metabolomic dataset for the clustering of related peaks with and without chromatographic peak shape correlations (see Section 3.4 of the Supplementary Material) shows that for best performance during the clustering stage, additional information, such as chromatographic peak shapes, should be used whenever available.

By looking at the testing performance, our results also explore the ability of the evaluated methods to generalize on different runs using less than optimal parameters. This is important because in the actual analytical situation of LC-MS data, neither the optimal parameters nor the alignment ground truth is known. The heterogeneous testing performance in the multiple-fractions experiment of P2 shows that no method performs best and the choice of optimal parameters that work for certain runs do not generalize well to others on datasets with very high RT variability. Using MW as an example, the optimal RT window parameter *σ_t_* is 90 seconds for training fraction 000 and 275 s for training fraction 080. We also observe that in the multiple-fractions experiment for P2, our proposed approach incorporating greedy clustering (MWG) shows a decrease in overall testing performance instead. This is because the greedy clustering method used is sensitive to the choice of parameters and do not generalize well across fractions of P2. The results suggest the dependence of our methods on the quality of groupings of related peaks in order to generalize well on different runs. The same conclusion can be obtained from the training and testing performances on the metabolomic dataset as well, where we see significant improvements in the training performance but none in the testing performance. On datasets with lower RT variation, such as the P1 and the glycomic data, we see evidence of improvements in both the training and testing performances, suggesting that incorporating clustering information in the proposed manner can indeed improve alignment performance and generalize well to different runs even with less than optimal parameter settings.

Note that our method relies on grouping of related peaks, and this introduces additional user-defined parameters. However, as our experiments have shown, in some settings, it may be much easier to produce good groupings of related peaks than accurately determine RT window parameters (the same grouping parameters were used for all evaluation datasets in the case of mixture-model clustering). Depending on the nature of the data, parameters relating to within-run characteristics (e.g. RT window for grouping related peaks) may be more likely to generalize across runs and experiments than parameters relating to between-run characteristics (particularly RT). For example, changes in the LC column would likely result in related peaks still coeluting but could significantly change the absolute RT.

It would be interesting to investigate in greater detail any performance improvements that can be obtained from using other peak grouping methods, such as Rogers *et al.* (2012) that uses a mixture model of peak shape correlations or Daly *et al.* (2014) that considers the dependencies between adduct and isotopic peaks when clustering. Exploring alternative approximate matching algorithms (such as the scaling algorithm in Duan *et al.* (2011), which provides a (1−ϵ) approximation of the maximum weighted matching in optimal linear time for any ϵ) and evaluating the benefits of incorporating different clustering approaches into our proposed alignment method are avenues for future work. Finally, the different alignment methods evaluated in this article also suffer from variable behaviors depending on the order of the runs being aligned. This is particularly true in the case of alignment of multiple runs (typical in large-scale LC-MS studies), where the final alignment results are often constructed through merging of intermediate alignments of pair-wise runs. Different alignment methods may employ a different merging approach, for example, Join merges the intermediate results towards a reference run while SIMA allows the possibility of using a greedy hierarchical merging scheme. Systematic evaluation on how the chosen merging scheme may influence alignment performance is beyond the scope of this article and is an item for future work.

The related peak-based similarity score that underpins our approach could be applied to many other direct matching approaches (e.g. SIMA: Voss *et al.*, 2011) and similar ideas could also be incorporated into recently developed methods that take into account the presence of internal standards (Tsai *et al.*, 2013). The evaluation pipeline developed over the course of our experiments can also be easily extended by algorithmic researchers to evaluate other alignment tools in future work.

## Funding

J.W. was funded by a PhD studentship from SICSA. R.D. was funded by an NWO Vidi fellowship grant to R.B. S.R and R.B. were supported by the BBSRC (BB/L018616/1).

*Conflict of Interest*: none declared.

## Supplementary Material

Supplementary Data
