# Supplementary material for: Incorporating peak grouping information for alignment of multiple liquid chromatography-mass spectrometry datasets
Source: Bioinformatics. 2015 Feb 2;31(12):1999–2006. doi: 10.1093/bioinformatics/btv072 (PMC4760236; doi:10.1093/bioinformatics/btv072)
Supplement: Supplementary Data [file supp_31_12_1999__index.html]

Incorporating peak grouping information for alignment of multiple liquid chromatography-mass spectrometry datasets — Incorporating peak grouping information for alignment of multiple liquid chromatography-mass spectrometry datasets — Incorporating peak grouping information for alignment of multiple liquid chromatography-mass spectrometry datasets — Supplementary Data 

# Incorporating peak grouping information for alignment of multiple liquid chromatography-mass spectrometry datasets

## Supplementary Data

files

**Files in this Data Supplement:**

- Supplementary Data - pdf file
