# Supplementary material for: Incorporating peak grouping information for alignment of multiple liquid chromatography-mass spectrometry datasets
Source: Bioinformatics. 2015 Feb 2;31(12):1999–2006. doi: 10.1093/bioinformatics/btv072 (PMC4760236; doi:10.1093/bioinformatics/btv072)
Supplement: Supplementary Data [file supp_btv072_supplementary.pdf]

Supplementary Materials

Incorporating peak grouping information for alignment of multiple liquid chromatography-mass spectrometry datasets

Joe Wandy[1], Rónán Daly[2], Rainer Breitling[3] and Simon Rogers[1]

January 5, 2015

[1], School of Computing Science, University of Glasgow, United Kingdom
[2] School of Computing & Mathematical Sciences, Liverpool John Moores University, United Kingdom
[3] Manchester Centre for Synthetic Biology of Fine and Speciality Chemicals (SYNBIOCHEM), Manchester Institute of Biotechnology, University of Manchester, United Kingdom

Contact: j.wandy.1@research.gla.ac.uk, r.daly@ljmu.ac.uk, rainer.breitling@manchester.ac.uk, simon.rogers@glasgow.ac.uk.

1 Dirichlet Process Mixture Model for RT Clustering

A peak feature refers to a tuple of $(m/z, RT)$ produced as output after the pre-processing of LC-MS data, where m/z is the mass-to-charge value and RT the retention time value of a peak feature. We can group related peaks together by RT. Our observation consists of a vector of N observed peak's RT values $\mathbf{y} = (y_1, y_2, ..., y_n)$. Our aim is to partition each set of peaks into K groups of related peaks (clusters) by their RT values. We used a Gaussian mixture model with Dirichlet Process prior (Rasmussen, 2000) to model the data. A peak is indexed by the variable $n = 1, ..., N$ and a cluster indexed by the variable $k = 1, ..., K$. Each Gaussian mixture component has some mean μ_k are assumed to have a fixed precision (inverse variance) δ, corresponding to the fixed retention time tolerance for each group of related peaks. Let the indicator $z_{nk} = 1$ denotes the assignment of peak n to RT cluster k. Then:

$$\boldsymbol{\pi}|\alpha \sim GEM(\gamma) \quad (1)$$
$$z_{nk} = 1|\boldsymbol{\pi}_k \sim \boldsymbol{\pi}_k \quad (2)$$
$$\mu_k|\mu_0, \tau_0 \sim \mathcal{N}(\mu_k|\mu_0, \tau_0^{-1}) \quad (3)$$
$$y_n|z_{nk} = 1, \mu_k \sim \mathcal{N}(\mu_k, \delta^{-1}) \quad (4)$$

where $\boldsymbol{\pi}$ is the mixing proportions, distributed according to the GEM (Griffiths, Engen and McCloskey) distribution. The GEM distribution over $\boldsymbol{\pi}$ is parameterised by the concentration parameter γ and is described through the stick-breaking construction:

$$\beta_k \sim Beta(1, \gamma) \tag{5}$$

$$\boldsymbol{\pi}_k = \beta_k \prod_{l=1}^{k-1} (1 - \beta_l) \tag{6}$$

The mixture component mean μ_k is drawn from a base Gaussian distribution with mean μ_0 and precision τ_0. We set μ_0 to the mean of the observed data, while τ_0 is set to a broad value of 5E-3. Analytical inference is not tractable here, so we use the Gibbs sampling scheme for inference. To do this, we need the conditional probability of $p(z_{nk} = 1, ...)$ of peak n to be in an existing cluster k (or k^* if a new cluster is to be created), given any other parameters in the model. This conditional probability is given by:

$$p(z_{nk} = 1 | \mathbf{y}_n, \ldots) \propto \begin{cases} c_k \cdot p(\mathbf{y}_n | z_{nk} = 1, ...) \\ \gamma \cdot p(\mathbf{y}_n | z_{nk^*} = 1, ...) \end{cases} \tag{7}$$

where c_k is the current number of members (peaks) in an existing cluster k. $p(\mathbf{y}_n | z_{nk} = 1, ...)$ is the likelihood of peak $\mathbf{y}_n$ in an existing cluster k. We can marginalise over all mixture components and get:

$$p(\mathbf{y}_n | z_{nk} = 1...) = \mathcal{N}(\mathbf{y}_n | \mu_k, \lambda_k^{-1}) \tag{8}$$

where $\lambda_k = ((\tau_0 + \sigma c_k)^{-1} + \delta^{-1})^{-1}$ and $\mu_k = \frac{1}{\lambda_k} [(\mu_0 \tau_0) + (\delta \sum_n \mathbf{y}_{n \in k})]$. Here, $\mathbf{y}_{n \in k}$ denotes the RT values of any peak n currently assigned to cluster k, and c_k the count of such peaks. The conditional probability of peak n to be in a new cluster k^* is:

$$p(\mathbf{y}_n | z_{nk^*} = 1...) = \mathcal{N}(\mathbf{y}_n | \mu_0, \lambda_{k^*}^{-1}) \tag{9}$$

where $\lambda_{k^*} = (\tau_0^{-1} + \sigma^{-1})^{-1}$.

In a step of the Gibbs sampling procedure, we perform the assignment of peak n to cluster k, creating new cluster k^* if necessary. For each sample, our primary interest is the marginal posterior of the probability of peak-vs-peak to be in the same cluster k. We obtain this using the posterior summaries across all samples drawn $S^* = \frac{1}{R} \sum_{r=1}^{R} s_r$, where s_r is the r-th posterior sample collected after a suitable burn-in period and R is the total number of samples taken (excluding burn-in samples). The result of this is a matrix of probabilities for any two peaks in the same run to be in the same cluster k, averaged over all samples.

2 Evaluation Datasets

Many direct matching methods work in a pairwise fashion and produce an overall results via some merging strategies of intermediate results. Pairwise performance therefore limits overall performance, and as such, we focus on evaluation using only pairs of runs. Some (P2, metabolomic and glycomic) of the datasets selected for evaluation in our experiments have more than 2 runs, so we select only 2 runs each to form a training and testing set. The procedure for doing so is described in the respective section for each dataset.

2.1 Proteomic Datasets

Lange et al. (2008) introduces two benchmark LC-MS proteomic sets (P1, P2) constructed to evaluate the ability of alignment tools in dealing with large retention time drifts, available from http://msbi.ipb-halle.de/msbi/caap and our site. Both the P1 and P2 datasets were analysed using an automated LC-LC/MS-MS platform. Each dataset comes in multiple chromatography salt-step fractions, obtained by bumping the salt level at every 10 minutes interval during chromatographic separation. P1 was produced from *E. coli* samples digested by trypsin, and comes in 2 runs for each fraction. P2 was obtained from *M. smegatis* protein extracts, similarly digested by trypsin, and contains 3 runs for each fraction. P2 was constructed to be a greater challenge to align with runs separated by weeks. Alignment ground truth is established in Lange et al. (2008) by means of peptides that can be reliably identified during the identification stage. Only identification annotations with SEQUEST Xcorr score >1.2 is included. Annotations are then filtered by their retention times and matched across runs.

For the proteomic datasets, each fraction in P1 has two runs used for alignment, while each fraction in P2 has three runs (we use only the first two to establish pairwise alignments). Tables 1 and 2 show the number of features for each run of the P1 and P2 datasets used for evaluations. Both P1 and P2 represent challenging alignment cases, with large deviations in RT values across runs. This is especially true for P2 with LC-MS runs separated by weeks and large differences in the number of features per run. Further details on the nature of the datasets can be found in Lange et al. (2008).

**Fraction**	**# runs**	**# features per run**
000	2	5824<br>4782
020	2	1114<br>1021
040	2	1230<br>958
060	2	1902<br>1440
080	2	1183<br>903
100	2	745<br>581

Table 1: No. of features in the P1 dataset

**Fraction**	**# runs**	**# features per run**
000	2	5054<br>5100
020	2	3271<br>529
040	2	1483<br>678
080	2	474<br>438
100	2	401<br>429

Table 2: No. of features in the P2 dataset

2.2 Metabolomic Datasets

We use a metabolomic dataset generated from a mixture of 104 standard metabolites used for the calibration of chromatographic columns (details in (Creek et al., 2011)). These runs were produced by ZIC-HILIC chromatography (Merck Sequant, Darmstadt, DE) on an UltiMate 3000 RSLC system (Thermo, Hemel Hempstead, UK), coupled to an Orbitrap Exactive mass spectrometer (Thermo, Hemel Hempstead, UK) in positive mode. The metabolomic dataset is available in different 11 runs, produced from different LC-MS

analyses separated by weeks. While these runs are not true technical replicates, they are similar enough to be treated as replicates for the purpose of performance evaluation, and they represent a realistic and fairly challenging alignment scenario. The output from each of these runs is available in PeakML format, which were then converted into a suitable format using the mzMatch suite (Scheltema et al., 2011). Both the original PeakML files and the converted text files can be found in our site.

Alignment ground truth was constructed from the putative identification of peaks in each of the 11 runs separately at 3 ppm using mzMatch's Identify module, taking as additional input a database of 104 compounds known to be present and a list of common adducts in positive ionisation mode (M+2H, M+H, M+ACN+H, 2M+Na, M+H+NH4, M+NH4, M+ACN+Na, 2M+ACN+H, M+ACN+2H, M+Na, M+2ACN+H, M+2ACN+2H, M +CH3OH+H, 2M+H). This is followed by matching of features that share same annotations across runs to construct the alignment ground truth. Only peaks unambiguously identified with exactly one annotation are used for this purpose, as peaks with more than one annotations per run are discarded from the ground truth construction. The results from this process is an alignment ground truth for a smaller subset of peaks in the runs that can be reliably identified at high mass precision.

The full metabolomic dataset comes in 11 runs in total. To generate the actual training and testing sets, 30 randomly pairs of runs were extracted as training sets, and another 30 pairs of runs extracted for testing sets. The following tables show the number of features in each run and the pairs of files selected as training and testing sets in our Metabolomic experiment.

**Filename**	**# features**	**Filename**	**# features**
std1-file1.txt	4999	std1-file7.txt	6319
std1-file2.txt	4986	std1-file8.txt	4101
std1-file3.txt	6836	std1-file9.txt	5485
std1-file4.txt	9752	std1-file10.txt	5034
std1-file5.txt	7076	std1-file11.txt	5317
std1-file6.txt	4146		

Table 3: No. of features in the full metabolomic dataset

Training Set	File 1	File 2	Training Set	File 1	File 2
**1**	std1-file9.txt	std1-file10.txt	**16**	std1-file11.txt	std1-file10.txt
**2**	std1-file2.txt	std1-file7.txt	**17**	std1-file3.txt	std1-file2.txt
**3**	std1-file11.txt	std1-file2.txt	**18**	std1-file7.txt	std1-file3.txt
**4**	std1-file6.txt	std1-file9.txt	**19**	std1-file7.txt	std1-file10.txt
**5**	std1-file6.txt	std1-file4.txt	**20**	std1-file9.txt	std1-file4.txt
**6**	std1-file10.txt	std1-file1.txt	**21**	std1-file1.txt	std1-file7.txt
**7**	std1-file9.txt	std1-file5.txt	**22**	std1-file7.txt	std1-file2.txt
**8**	std1-file8.txt	std1-file1.txt	**23**	std1-file6.txt	std1-file1.txt
**9**	std1-file10.txt	std1-file2.txt	**24**	std1-file9.txt	std1-file4.txt
**10**	std1-file11.txt	std1-file1.txt	**25**	std1-file3.txt	std1-file7.txt
**11**	std1-file9.txt	std1-file3.txt	**26**	std1-file5.txt	std1-file9.txt
**12**	std1-file8.txt	std1-file9.txt	**27**	std1-file11.txt	std1-file2.txt
**13**	std1-file2.txt	std1-file8.txt	**28**	std1-file10.txt	std1-file1.txt
**14**	std1-file11.txt	std1-file4.txt	**29**	std1-file1.txt	std1-file5.txt
**15**	std1-file3.txt	std1-file9.txt	**30**	std1-file2.txt	std1-file3.txt

Table 4: Selections of 30 pairs of files used as training sets in the Metabolomic experiment.

Testing Set	File 1	File 2	Testing Set	File 1	File 2
**1**	std1-file6.txt	std1-file4.txt	**16**	std1-file5.txt	std1-file4.txt
**2**	std1-file9.txt	std1-file2.txt	**17**	std1-file3.txt	std1-file9.txt
**3**	std1-file2.txt	std1-file5.txt	**18**	std1-file9.txt	std1-file6.txt
**4**	std1-file7.txt	std1-file11.txt	**19**	std1-file11.txt	std1-file6.txt
**5**	std1-file4.txt	std1-file10.txt	**20**	std1-file4.txt	std1-file8.txt
**6**	std1-file8.txt	std1-file2.txt	**21**	std1-file6.txt	std1-file10.txt
**7**	std1-file5.txt	std1-file7.txt	**22**	std1-file11.txt	std1-file2.txt
**8**	std1-file3.txt	std1-file8.txt	**23**	std1-file6.txt	std1-file1.txt
**9**	std1-file7.txt	std1-file9.txt	**24**	std1-file8.txt	std1-file9.txt
**10**	std1-file6.txt	std1-file9.txt	**25**	std1-file5.txt	std1-file10.txt
**11**	std1-file4.txt	std1-file6.txt	**26**	std1-file7.txt	std1-file8.txt
**12**	std1-file8.txt	std1-file9.txt	**27**	std1-file11.txt	std1-file1.txt
**13**	std1-file3.txt	std1-file7.txt	**28**	std1-file5.txt	std1-file3.txt
**14**	std1-file3.txt	std1-file10.txt	**29**	std1-file7.txt	std1-file5.txt
**15**	std1-file5.txt	std1-file11.txt	**30**	std1-file11.txt	std1-file4.txt

Table 5: Selections of 30 pairs of files used as testing sets in the Metabolomic experiment.

2.3 Glycomic Dataset

Tsai et al. (2013) provides a glycomic dataset containing 23 runs, available from http://omics.georgetown.edu/alignLCMS.html and our site. The glyomic dataset were produced from untargeted LC-MS study for identifying N-glycan disease biomarkers. LC-MS data were acquired from a Dionex 3000 Ultimate nano-LC system, coupled to an LTQ-Orbitrap Velos mass spectrometer on positive mode. Alignment ground truth is established in Tsai et al. (2013) based on a manual comparison of measured mass values with theoretical values (taking into account hydrogen adducts) and visual inspection of potentially incorrect assignments.

We randomly extracted 30 pairs of runs for training and another 30 pairs of runs for testing performance evaluation from the full glyocomic dataset provided by Tsai et al. (2013), which comes in 23 runs in total. The following tables show the number of features in each run and the indices of the pairs of files randomly selected as training and testing sets in our Glycomic experiment.

**File index**	**Filename**	**# features**	**File index**	**Filename**	**# features**
0	G1_1.txt	856	12	G1_13.txt	911
1	G1_2.txt	1088	13	G1_14.txt	1144
2	G1_3.txt	922	14	G1_15.txt	932
3	G1_4.txt	808	15	G1_16.txt	1541
4	G1_5.txt	886	16	G1_17.txt	1022
5	G1_6.txt	850	17	G1_18.txt	1051
6	G1_7.txt	979	18	G1_19.txt	1119
7	G1_8.txt	1008	19	G1_20.txt	1047
8	G1_9.txt	904	20	G1_21.txt	1017
9	G1_10.txt	1043	21	G1_22.txt	990
10	G1_11.txt	1041	22	G1_23.txt	977
11	G1_12.txt	885			

Table 6: No. of features in the full glycomic dataset from Tsai et al. (2013)

Training Set	File 1	File 2	Training Set	File 1	File 2
**1**	G1_19.txt	G1_21.txt	**16**	G1_12.txt	G1_17.txt
**2**	G1_3.txt	G1_15.txt	**17**	G1_10.txt	G1_11.txt
**3**	G1_23.txt	G1_4.txt	**18**	G1_20.txt	G1_6.txt
**4**	G1_12.txt	G1_19.txt	**19**	G1_9.txt	G1_22.txt
**5**	G1_2.txt	G1_1.txt	**20**	G1_3.txt	G1_9.txt
**6**	G1_20.txt	G1_1.txt	**21**	G1_14.txt	G1_13.txt
**7**	G1_18.txt	G1_10.txt	**22**	G1_18.txt	G1_9.txt
**8**	G1_17.txt	G1_1.txt	**23**	G1_2.txt	G1_13.txt
**9**	G1_19.txt	G1_3.txt	**24**	G1_14.txt	G1_3.txt
**10**	G1_22.txt	G1_1.txt	**25**	G1_8.txt	G1_4.txt
**11**	G1_18.txt	G1_19.txt	**26**	G1_13.txt	G1_4.txt
**12**	G1_2.txt	G1_8.txt	**27**	G1_18.txt	G1_16.txt
**13**	G1_6.txt	G1_10.txt	**28**	G1_6.txt	G1_22.txt
**14**	G1_12.txt	G1_3.txt	**29**	G1_13.txt	G1_23.txt
**15**	G1_14.txt	G1_6.txt	**30**	G1_3.txt	G1_23.txt

Table 7: Selections of 30 pairs of files used as training sets in the Glycomic experiment.

Testing Set	File 1	File 2	Testing Set	File 1	File 2
**1**	G1_19.txt	G1_20.txt	**16**	G1_5.txt	G1_16.txt
**2**	G1_6.txt	G1_19.txt	**17**	G1_9.txt	G1_4.txt
**3**	G1_7.txt	G1_5.txt	**18**	G1_12.txt	G1_11.txt
**4**	G1_20.txt	G1_14.txt	**19**	G1_19.txt	G1_12.txt
**5**	G1_4.txt	G1_12.txt	**20**	G1_18.txt	G1_3.txt
**6**	G1_10.txt	G1_2.txt	**21**	G1_21.txt	G1_2.txt
**7**	G1_6.txt	G1_5.txt	**22**	G1_5.txt	G1_14.txt
**8**	G1_22.txt	G1_21.txt	**23**	G1_6.txt	G1_20.txt
**9**	G1_21.txt	G1_8.txt	**24**	G1_6.txt	G1_4.txt
**10**	G1_9.txt	G1_18.txt	**25**	G1_7.txt	G1_9.txt
**11**	G1_3.txt	G1_4.txt	**26**	G1_11.txt	G1_21.txt
**12**	G1_10.txt	G1_5.txt	**27**	G1_10.txt	G1_14.txt
**13**	G1_6.txt	G1_8.txt	**28**	G1_6.txt	G1_17.txt
**14**	G1_17.txt	G1_5.txt	**29**	G1_10.txt	G1_8.txt
**15**	G1_13.txt	G1_7.txt	**30**	G1_7.txt	G1_19.txt

Table 8: Selections of 30 pairs of files used as testing sets in the Glycomic experiment.

3 Parameter Optimisations, Running Time and Detailed Results

For every evaluated method in our experiments, we performed grid-search on the m/z and RT windows parameters using the training set. We then used those optimal parameters to perform alignment on the testing set, giving us the respective performance measures (Precision, Recall, F_1) on the testing set. For testing set consisting of multiple fractions, we report the average performance measures on the testing fractions.

For training using the P1 and P2 datasets in the proteomic experiments, the m/z and RT tolerances were varied within: $\{1.0, 1.2, ..., 2.0\}$ for the m/z tolerance, and $\{5, 10, .., 300\}$ seconds for the RT tolerance. The parameter ranges were chosen based on reasonable estimates of the instrument's precision and prior RT tolerance values as reported by Lange et al. (2008). We kept all the default values for the remaining parameters in each evaluated tool, if any. For MWG, we also varied the ratio parameter α from $\{0.1, 0.2, ..., 1.0\}$ and the grouping parameter g_{tol} from $\{1, 2, ..., 10\}$ seconds and uses the combination that results in the best performance. For MWM, the ratio parameter α was varied from $\{0.1, 0.2, ..., 1.0\}$ but mixture model parameters were kept the same for clustering of all fractions in P1 and P2. When clustering all fractions in a dataset, a broad Gaussian prior was set for the component mean μ_j of each cluster j. The component precision s_j was set to 5 seconds, while the DP concentration parameter γ is set to 1. We drew 2000 posterior samples (with 1000 initial burn-in samples) for each run during the Gibbs sampling steps to construct the probability matrix of peak-vs-peak to be in the same cluster.

For the Metabolomic and Glygomic experiments, 30 pairs of run were randomly extracted from the M1 metabolomic dataset in Lange et al. (2008) and from the glycomic dataset in Tsai et al. (2013). These were assigned to be the training sets. Another 30 pairs of runs were extracted from each dataset to be the testing sets. Each pair of runs in the training set is assigned a partner pair of runs in the testing set. Parameters were optimised on pairwise runs in the training set and performance evaluated on the assigned partner runs in the testing set. For both datasets, the m/z tolerances used were $\{0.05, 0.1, 0.25\}$ and RT $\{5, 10, 15, ...100\}$ seconds. These ranges of parameters were selected in view of instrument accuracy and RT noise level of the LC-MS instruments that generate our metabolomic data and in Tsai et al. (2013). The ratio parameter α was from $\{0.1, 0.2, ..., 1.0\}$ and the grouping parameter g_{tol} from $\{2, 4, ..., 10\}$ seconds for both datasets, and for the metabolomic dataset where chromatographic peak shapes information is available and used for greedy clustering in MWG, the threshold for the Pearson correlation coefficient between peak shape signals was varied from $c = \{0.70, 0.75, 0.80, 0.85, 0.90, 0.95\}$.

3.1 Running Time

Computational times of the proposed methods are primarily affected by the number of features in the runs being aligned and to some extent, the thresholding parameters

used during similarity score computation and feature matching. Table 9 reports the measured running time for each proposed method using the parameters that give the best training performance (in the results of Table 10). For each fraction being aligned, the running times were measured three times on a standard laptop with Intel Core i5 CPU running at 2.5 GHz, and the average value reported for matching only (MW), matching incorporating greedy clustering (MWG) and matching incorporating mixture model clustering (MWM). The time complexity of the mixture-model clustering step in MWM is $O(N)$ where N is the number of features in the run being clustered. We took 2000 posterior samples, discarding the first 1000 samples during the burn-in period. The number of samples were chosen to ensure convergence to the stationary distribution during inference procedure. Further details on parameters for each method can be found in Section 3.2.

**Fraction**	**# runs**	**# features per run**	**MW**	**MWG**	**MWM**
000	2	5824<br>4782	8.6 s	11.6 s	2669.6 s
020	2	1114<br>1021	1.3 s	1.7 s	524.1 s
040	2	1230<br>958	1.6 s	2.1 s	540.1 s
060	2	1902<br>1440	2.1 s	2.7 s	824.7 s
080	2	1183<br>903	1.6 s	2.0 s	504.7 s
100	2	745<br>581	1.2 s	1.5 s	320.7 s

Table 9: Running time on the P1 dataset

3.2 Single-fraction Experiments Results

This section reports the optimal performance (as measured by Precision, Recall and the F_1-scores) on the proteomic datasets (P1, P2) first introduced in Lange et al. (2008).

**Training Fraction**	**Measure**	**Join**	**SIMA**	**MW**	**MWG**	**MWM**
000	Prec	0.61	0.62	0.62	0.75	0.68
	Rec	0.66	0.66	0.67	0.79	0.74
	F_1	0.63	0.64	0.64	**0.77**	0.71
020	Prec	0.92	0.92	0.92	0.95	0.94
	Rec	0.85	0.85	0.85	0.95	0.85
	F_1	0.88	0.88	0.88	**0.95**	0.90
040	Prec	0.83	0.85	0.86	0.89	0.87
	Rec	0.81	0.81	0.83	0.86	0.85
	F_1	0.82	0.83	0.85	**0.87**	0.86
060	Prec	0.74	0.75	0.75	0.86	0.81
	Rec	0.78	0.80	0.80	0.90	0.86
	F_1	0.76	0.78	0.78	**0.88**	0.83
080	Prec	0.90	0.89	0.88	0.92	0.90
	Rec	0.89	0.89	0.89	0.92	0.90
	F_1	0.90	0.89	0.88	**0.92**	0.90
100	Prec	0.88	0.88	0.88	0.90	0.90
	Rec	0.90	0.90	0.90	0.92	0.92
	F_1	0.89	0.89	0.89	**0.91**	**0.91**
**Mean**	F_1	0.81	0.82	0.82	**0.88**	0.85

Table 10: Full results for the single-fraction experiment results for P1 (Table 1 in the paper). The tool with the highest F_1 score for each fraction is highlighted in bold.

Training Fraction	Join	SIMA	MW	MWG	MWM
000	m/z tolerance = 1.5<br>RT tolerance = 100	$T_{(m/z)}$= 1.7<br>$T_{(rt)}$ = 100	σ_m = 1.7<br>σ_t = 100	σ_m = 1.7<br>σ_t = 105<br>$\alpha = 0.2$<br>g_{tol} = 10	σ_m = 1.4<br>σ_t = 135<br>$\alpha = 0.1$<br>$\delta = 0.2$<br>$\gamma = 1$
020	m/z tolerance = 1.4<br>RT tolerance = 105	$T_{(m/z)}$= 1.4<br>$T_{(rt)}$ = 115	σ_m = 1.4<br>σ_t = 110	σ_m = 1.5<br>σ_t = 285<br>$\alpha = 0.3$<br>g_{tol} = 9	σ_m = 1.3<br>σ_t = 70<br>$\alpha = 0.1$<br>$\delta = 0.2$<br>$\gamma = 1$
040	m/z tolerance = 1.4<br>RT tolerance = 90	$T_{(m/z)}$= 1.6<br>$T_{(rt)}$ = 90	σ_m = 1.6<br>σ_t = 90	σ_m = 1.4<br>σ_t = 90<br>$\alpha = 0.3$<br>g_{tol} = 4	σ_m = 1.4<br>σ_t = 90<br>$\alpha = 0.1$<br>$\delta = 0.2$<br>$\gamma = 1$
060	m/z tolerance = 1.4<br>RT tolerance = 160	$T_{(m/z)}$= 1.7<br>$T_{(rt)}$ = 185	σ_m = 1.5<br>σ_t = 170	σ_m = 1.4<br>σ_t = 150<br>$\alpha = 0.2$<br>g_{tol} = 9	σ_m = 1.5<br>σ_t = 145<br>$\alpha = 0.1$<br>$\delta = 0.2$<br>$\gamma = 1$
080	m/z tolerance = 1.4<br>RT tolerance = 300	$T_{(m/z)}$= 1.6<br>$T_{(rt)}$ = 250	σ_m = 1.5<br>σ_t = 285	σ_m = 1.5<br>σ_t = 220<br>$\alpha = 0.5$<br>g_{tol} = 6	σ_m = 1.5<br>σ_t = 215<br>$\alpha = 0.2$<br>$\delta = 0.2$<br>$\gamma = 1$
100	m/z tolerance = 1.6<br>RT tolerance = 145	$T_{(m/z)}$= 1.5<br>$T_{(rt)}$ = 140	σ_m = 2.0<br>σ_t = 220	σ_m = 1.4<br>σ_t = 220<br>$\alpha = 0.3$<br>g_{tol} = 3	σ_m = 1.4<br>σ_t = 75<br>$\alpha = 0.1$<br>$\delta = 0.2$<br>$\gamma = 1$

Table 11: Single-fraction optimal training parameters for P1.

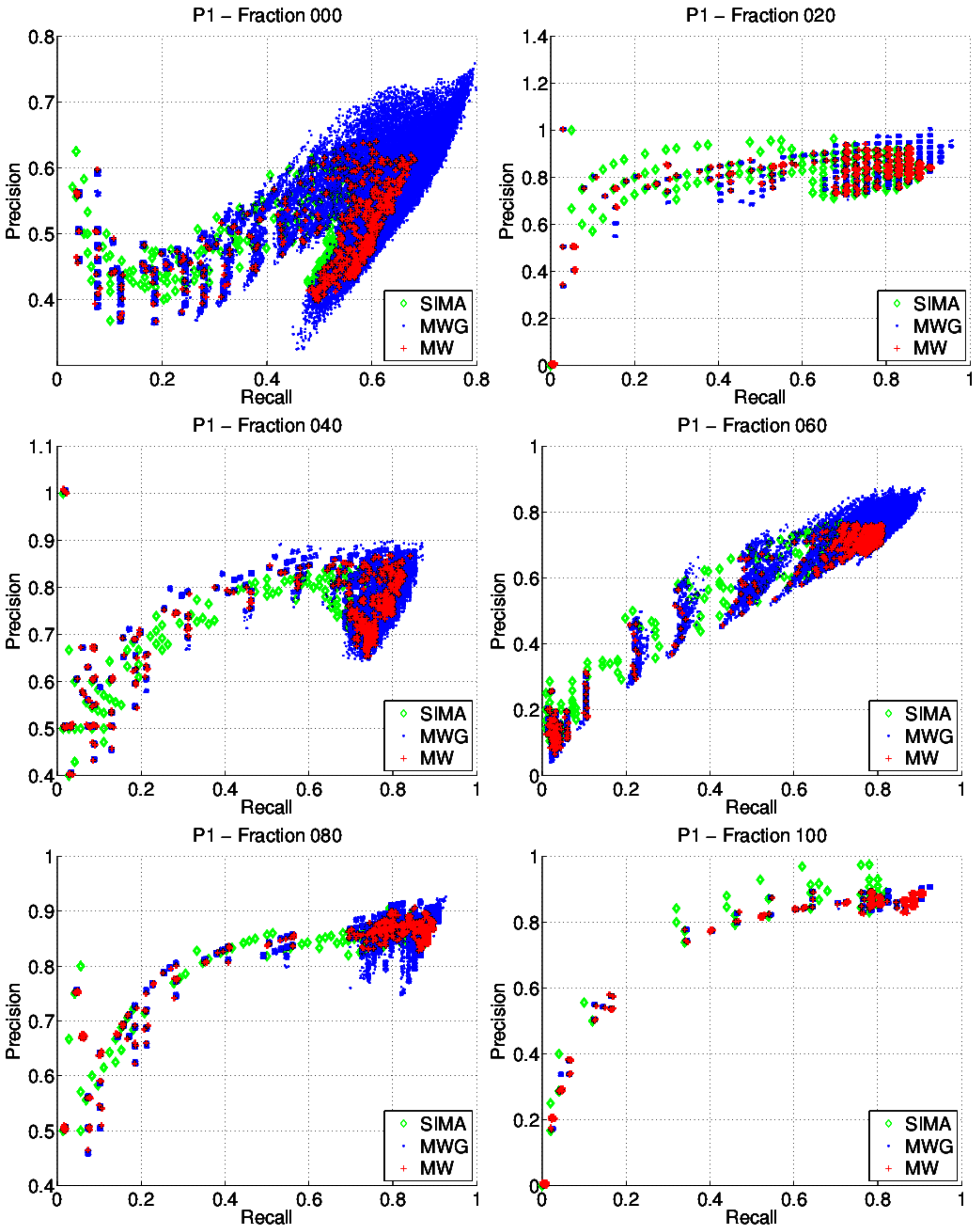

Figure 1: Full results for the P1 training performance of every possible m/z and RT tolerance parameters (also shown in Figure 2 in the paper). For MWG, the ratio parameter α and the grouping tolerance g_{tol} are varied as well (resulting in more data points).

**Training Fraction**	**Measure**	**Join**	**SIMA**	**MW**	**MWG**	**MWM**
000	Prec	0.44	0.44	0.44	0.49	0.44
	Rec	0.46	0.46	0.46	0.48	0.46
	F_1	0.45	0.45	0.45	**0.49**	0.45
020	Prec	0.76	0.77	0.78	0.79	0.78
	Rec	0.78	0.80	0.80	0.81	0.80
	F_1	0.77	0.78	0.79	**0.80**	0.79
040	Prec	0.75	0.77	0.75	0.78	0.75
	Rec	0.79	0.79	0.79	0.82	0.79
	F_1	0.77	0.78	0.77	**0.80**	0.77
080	Prec	0.66	0.70	0.68	0.68	0.72
	Rec	0.66	0.66	0.66	0.66	0.72
	F_1	0.66	0.68	0.67	0.67	**0.72**
100	Prec	0.55	0.61	0.58	0.85	0.65
	Rec	0.55	0.55	0.55	0.85	0.75
	F_1	0.55	0.58	0.56	**0.85**	0.70
**Mean**	F_1	0.64	0.65	0.65	**0.72**	0.69

Table 12: Full results for the single-fraction experiment results for P2 (Table 2 in the paper). The tool with the highest F_1 score for each fraction is highlighted in bold.

**Training Fraction**	**Join**	**SIMA**	**MW**	**MWG**	**MWM**
000	m/z tolerance = 1.3<br>RT tolerance = 115	$T_{(m/z)}$= 1.4<br>$T_{(rt)}$ = 135	σ_m = 1.4<br>σ_t = 90	σ_m = 1.1<br>σ_t = 115<br>$\alpha = 0.2$<br>g_{tol} = 5	σ_m = 1.4<br>σ_t = 90<br>$\alpha = 1$<br>$\delta = 0.2$<br>$\gamma = 1$
020	m/z tolerance = 1.5<br>RT tolerance = 125	$T_{(m/z)}$= 1.8<br>$T_{(rt)}$ = 155	σ_m = 1.4<br>σ_t = 120	σ_m = 1.4<br>σ_t = 120<br>$\alpha = 0.3$<br>g_{tol} = 7	σ_m = 1.4<br>σ_t = 120<br>$\alpha = 0.9$<br>$\delta = 0.2$<br>$\gamma = 1$
040	m/z tolerance = 1.7<br>RT tolerance = 105	$T_{(m/z)}$= 1.7<br>$T_{(rt)}$ = 145	σ_m = 1.6<br>σ_t = 140	σ_m = 1.8<br>σ_t = 160<br>$\alpha = 0.2$<br>g_{tol} = 3	σ_m = 1.5<br>σ_t = 130<br>$\alpha = 0.9$<br>$\delta = 0.2$<br>$\gamma = 1$
080	m/z tolerance = 1.5<br>RT tolerance = 225	$T_{(m/z)}$= 1.2<br>$T_{(rt)}$ = 300	σ_m = 1.1<br>σ_t = 275	σ_m = 1.1<br>σ_t = 275<br>$\alpha = 0.1$<br>g_{tol} = 1	σ_m = 1.8<br>σ_t = 195<br>$\alpha = 0.7$<br>$\delta = 0.2$<br>$\gamma = 1$
100	m/z tolerance = 1.7<br>RT tolerance = 140	$T_{(m/z)}$= 1.51<br>$T_{(rt)}$ = 115	σ_m = 1.0<br>σ_t = 140	σ_m = 1.6<br>σ_t = 215<br>$\alpha = 0.2$<br>g_{tol} = 8	σ_m = 1.3<br>σ_t = 250<br>$\alpha = 0.1$<br>$\delta = 0.2$<br>$\gamma = 1$

Table 13: Single-fraction optimal training parameters for P2.

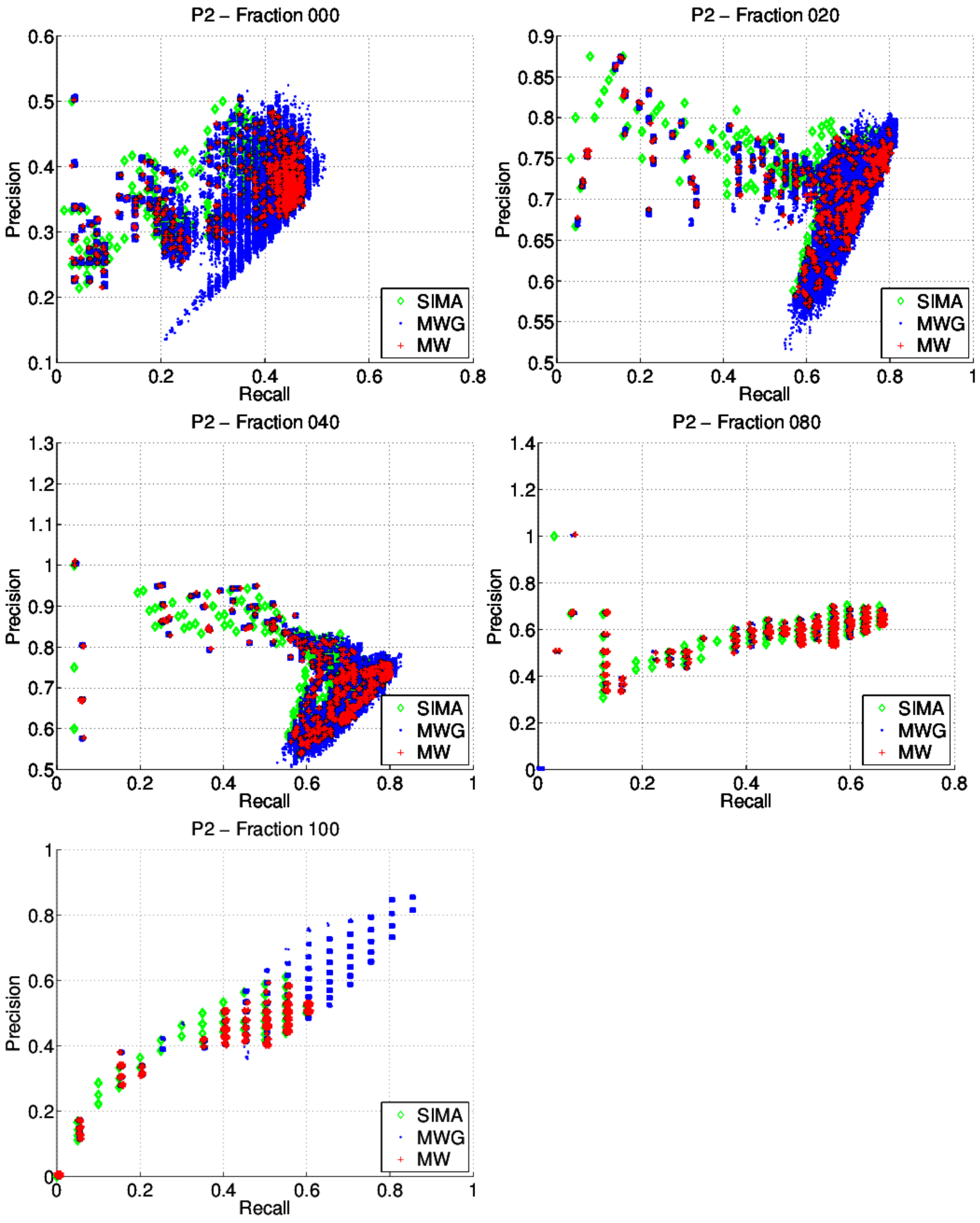

Figure 2: Full results for the P2 training performance of every possible m/z and RT tolerance parameters (also shown in Figure 2 in the paper). For MWG, the ratio parameter α and the grouping tolerance g_{tol} are varied as well (resulting in more data points).

3.3 Multiple-fractions Experiments Results

This section reports the testing performance (as measured by Precision, Recall and the F_1-scores) on the proteomic datasets (P1, P2) first introduced in Lange et al. (2008). For each dataset, a training fraction is selected and used to obtain the optimal parameters (as shown in Tables 11 and 13). Performance is then evaluated using these optimal parameters on the testing fractions, which are the remaining fractions in the dataset.

**Training Fraction**	**Testing Performance**					
	**Measure**	**Join**	**SIMA**	**MW**	**MWG**	**MWM**
000	Prec	0.82	0.86	0.82	0.86	0.86
	Rec	0.82	0.85	0.83	0.86	0.86
	F_1	0.82	0.85	0.82	**0.86**	**0.86**
020	Prec	0.77	0.76	0.77	0.77	0.79
	Rec	0.79	0.75	0.79	0.81	0.71
	F_1	0.78	0.76	0.78	**0.79**	0.75
040	Prec	0.79	0.77	0.77	0.80	0.81
	Rec	0.78	0.75	0.78	0.79	0.81
	F_1	0.78	0.76	0.77	0.79	**0.81**
060	Prec	0.77	0.76	0.76	0.83	0.82
	Rec	0.79	0.80	0.79	0.84	0.84
	F_1	0.78	0.78	0.77	**0.84**	0.83
080	Prec	0.68	0.71	0.69	0.74	0.76
	Rec	0.75	0.76	0.76	0.80	0.80
	F_1	0.71	0.73	0.72	0.77	**0.78**
100	Prec	0.74	0.77	0.71	0.74	0.81
	Rec	0.77	0.77	0.78	0.78	0.76
	F_1	0.75	0.77	0.74	0.76	**0.78**
**Mean**	Prec	0.76	0.77	0.75	0.79	0.81
	Rec	0.78	0.78	0.79	0.81	0.80
	F_1	0.77	0.78	0.77	**0.80**	**0.80**

Table 14: Full results for the multiple-fractions experiment for P1 (Table 3 in the paper). For each training fraction, the reported testing performance is the average of individual F_1 scores from the testing fractions (all other fractions in the dataset, except the training fraction). The top-performing method (highest F_1 score) is highlighted in bold.

**Training Fraction**	**Testing Performance**					
	**Measure**	**Join**	**SIMA**	**MW**	**MWG**	**MWM**
000	Prec	0.64	0.66	0.63	0.50	0.63
	Rec	0.60	0.62	0.59	0.46	0.59
	F_1	0.62	**0.64**	0.61	0.48	0.61
020	Prec	0.58	0.54	0.55	0.43	0.55
	Rec	0.59	0.58	0.54	0.43	0.54
	F_1	**0.58**	0.56	0.55	0.43	0.55
040	Prec	0.53	0.55	0.55	0.38	0.56
	Rec	0.52	0.57	0.58	0.43	0.56
	F_1	0.52	**0.56**	**0.56**	0.41	**0.56**
080	Prec	0.53	0.48	0.48	0.48	0.54
	Rec	0.60	0.52	0.52	0.52	0.62
	F_1	0.56	0.50	0.50	0.50	**0.57**
100	Prec	0.60	0.61	0.59	0.42	0.55
	Rec	0.65	0.54	0.53	0.46	0.59
	F_1	**0.63**	0.57	0.56	0.44	0.57
**Mean**	Prec	0.58	0.57	0.56	0.44	0.57
	Rec	0.59	0.57	0.56	0.46	0.58
	F_1	**0.58**	0.57	0.56	0.45	0.57

Table 15: Full results for the multiple-fractions experiment for P1 (Table 4 in the paper). For each training fraction, the reported testing performance is the average of individual F_1 scores from the testing fractions (all other fractions in the dataset, except the training fraction). The top-performing method (highest F_1 score) is highlighted in bold.

3.4 Metabolomic Experiments Results

We also compared the results for MWG on both the training and testing sets on the standard metabolomic dataset when the greedy grouping is performed using only RT information (MWG (RT)) and when chromatographic peak shape correlations are also considered (MWG(RT+PS)) during the grouping process. The results for MWG shown in the main paper corresponds to the results for MWG(RT+PS) here.

Statistically significant differences can be observed on the training performance of Figure 3, with the mean of F_1 scores for MW 0.83, MWG(RT) 0.88 and MWG(RT+PS) 0.90. However, this does not translate to any improvements on the testing sets, with the mean of F_1 scores for MW 0.86, MWG(RT) 0.83 and MWG(RT+PS) 0.85. Introducing clustering information when only RT information is used during the clustering process (MWG(RT)) reduces testing performance. The training results suggest that where additional information such as chromatographic peak shapes are available, they should be used for the clustering step in the proposed methods. However, the lack of any statistically significant testing improvements between MW and MWG (RT+PS), suggest that the optimal parameters from training runs do not generalise well to different testing runs for the greedy clustering approach in general, especially for complex metabolomic runs, with large number of features that tend to closely co-elute with each other.

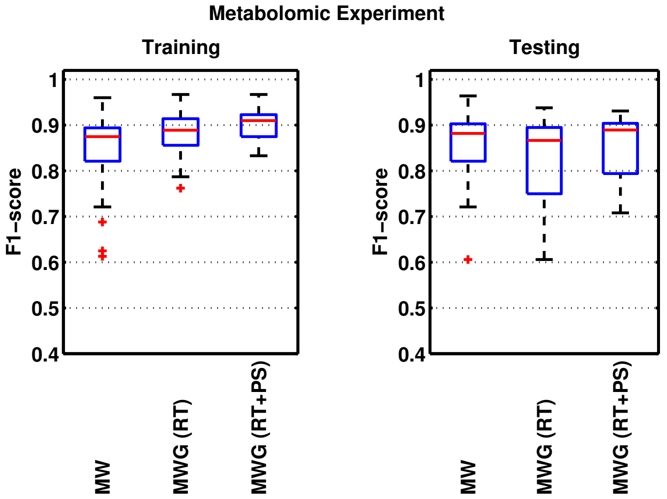

Figure 3: Comparisons in matching performance when greedy clustering with retention time (MWG(RT)) and peak shape correlations (MWG(RT+PS)) are used.

References

Darren J Creek, Andris Jankevics, Rainer Breitling, David G Watson, Michael P Barrett, and Karl E V Burgess. Toward global metabolomics analysis with hydrophilic interaction liquid chromatography–mass spectrometry: improved metabolite identification by retention time prediction. *Analytical Chemistry*, 83(22):8703–8710, 2011.

Eva Lange, Ralf Tautenhahn, Steffen Neumann, and Clemens Gröpl. Critical assessment of alignment procedures for {LC}-{MS} proteomics and metabolomics measurements. *BMC Bioinformatics*, 9:375, 2008.

Carl Edward Rasmussen. The Infinite {G}aussian Mixture Model. In *Advances in Neural Information Processing Systems 12*, pages 554–560. MIT Press, 2000.

Richard a Scheltema, Andris Jankevics, Ritsert C Jansen, Morris a Swertz, and Rainer Breitling. PeakML/mzMatch: a file format, Java library, R library, and tool-chain for mass spectrometry data analysis. *Analytical Chemistry*, 83(7): 2786–93, 2011.

Tsung-Heng Tsai, Mahlet G Tadesse, Cristina Di Poto, Lewis K Pannell, Yehia Mechref, Yue Wang, and Habtom W Ressom. Multi-profile Bayesian alignment model for LC-MS data analysis with integration of internal standards. *Bioinformatics*, 29(21):2774–80, 2013.
